# Bioactive Hydantoin Alkaloids from the Red Sea Marine Sponge *Hemimycale arabica*

**DOI:** 10.3390/md13116609

**Published:** 2015-10-28

**Authors:** Diaa T. A. Youssef, Lamiaa A. Shaala, Khalid Z. Alshali

**Affiliations:** 1 Department of Natural Products, Faculty of Pharmacy, King Abdulaziz University, Jeddah 21589, Saudi Arabia; 2 Natural Products Unit, King Fahd Medical Research Center, King Abdulaziz University, Jeddah 21589, Saudi Arabia; E-Mail: lshalla@kau.edu.sa; 3 Suez Canal University Hospital, Suez Canal University, Ismailia 41522, Egypt; 4 Department of Medicine, Faculty of Medicine, King Abdulaziz University, Jeddah 21589, Saudi Arabia; E-Mail: kalshali@kau.edu.sa or kzalshal@yahoo.com

**Keywords:** Red Sea sponge, *Hemimycale arabica*, *N-*alkylated hydantoins, hemimycalins A and B, antimicrobial and antiproliferative activities, HeLa cells

## Abstract

In the course of our continuing efforts to identify bioactive secondary metabolites from Red Sea marine invertebrates, we have investigated the sponge *Hemimycale* *arabica*. The antimicrobial fraction of an organic extract of the sponge afforded two new hydantoin alkaloids, hemimycalins A and B (**2** and **3**), together with the previously reported compound (*Z*)-5-(4-hydroxybenzylidene)imidazolidine-2,4-dione (**1**). The structures of the compounds were determined by extensive 1D and 2D NMR (COSY, HSQC and HMBC) studies and high-resolution mass spectral determinations. Hemimycalins A (**2**) and B (**3**) represent the first examples of the natural *N*-alkylated hydantoins from the sponge *Hemimycale arabica*. Compounds **1**–**3** displayed variable antimicrobial activities against *E. coli*, *S. aureus*, and *C. albicans*. In addition, compound **1** displayed moderate antiproliferative activity against the human cervical carcinoma (HeLa) cell line. These findings provide further insight into the chemical diversity as well as the biological activity of this class of compounds.

## 1. Introduction

The marine environment has proven to be a very rich source of extremely potent compounds with significant activities including antitumor, anti-inflammatory, analgesic, immunomodulatory, anti-allergic, and anti-viral [1]. Bioactive natural products have been isolated from marine macro- or micro-organisms. To date, about 24,662 new compounds have been reported from marine organisms since 1963 [2]. Marine sponges (phylum Porifera) are among the oldest multicellular invertebrate organisms [3], exhibiting a wide variety of colors and shapes. To date, about 11,000 species have been formally described, of which approximately 8500 are considered valid [4]. Marine sponges continue to attract wide attention from marine natural product chemists and pharmacologists alike due to their remarkable diversity of bioactive compounds [5,6]. A total of 10 invertebrate-derived compounds are currently in advanced phases of clinical trials [6]. This fact clearly demonstrates and reflects the importance of marine sponges as a potential source for future drug discovery and development [6]. Members of the genus *Hemimycale* are producers of bioactive secondary metabolites including the complex guanidine alkaloids [7,8] and hydantoin derivatives [9]. Examples of these compounds include ptilomycalin A. It possesses the unique polycyclic guanidine moiety connected with a ω-hydroxyhexadecanoyl-spermidine group through an ester linkage [7,8]. This guanidine alkaloid showed remarkable antifungal, antiviral, and antitumor activities [7,8].

Hydantoin (1,3-imidazolidinedione) derivatives display diverse and interesting pharmacological properties. Several such derivatives (phenytoin, mephenythoin, norantoin, methetoin, ethotoin, fosphenytoin) are well-known anticonvulsive drugs [9,10]. Other 5-substituted hydantoins like 5,5-dithienylhydantoin, 5,5-dipyridylhydantoin, spirothiohydantoin, thiohydantoin, and dithiohydantoins also possess anticonvulsive activity [11,12]. Hydantoin derivatives can also be found as antiarrhythmics (azimilide), antimicrobial agents (nitrofurantoin), skeletal muscle relaxants (dantrolene), and nonsteroidal antiandrogens (nilutamide), while allantoin is used as a keratolytic, astringent, wound remedy, antacid, and antipsoriatic drug [10]. Hydantoins also exhibit antidepressant, antiviral, and antithrombotic activities, as well as inhibitory activity against some enzymes (human aldose reductase and human leucocyte elastase) [13]. Finally, some herbicides (spirohydantoin, thioxohydantocidin), fungicides (clodantoin), and insecticides also have the hydantoin skeleton in their structure [14,15]. Some spirohydantoins are considered to be a novel aldose reductase inhibitor to treat diabetes [16].

The natural (*Z*)-5-(4-hydroxybenzylidene) imidazolidine-2,4-dione (**1**) and its synthetic derivative (*Z*)-5-(4-(ethylthio)benzylidene)-hydantoin showed potent *in vitro* anti-growth and anti-invasive properties against PC-3M prostate cancer cells in MTT and spheroid disaggregation [17]. They decreased the orthotopic tumor growth and inhibited the formation of tumor micrometastases in distant organs without apparent cytotoxic effects at the test doses [17].

In the course of our ongoing search for bioactive compounds from Red Sea marine sponges, we have investigated the antimicrobial fraction of an organic extract of the Red Sea sponge *Hemimycale arabica*. The study resulted in the identification of three hydantoin alkaloids including two new compounds, hemimycalyins A and B (**2** and **3**), and the previously reported compound (*Z*)-5-(4-hydroxybenzylidene) imidazolidine-2,4-dione (**1**) [18,19]. In this paper, the purification, structure determination of compounds **1**–**3** as well as the antimicrobial and antiproliferative activities of the compounds will be discussed.

## 2. Results and Discussion

### 2.1. Purification of Compounds **1**–**3**

Chromatographic investigation of the organic extract of the Red Sea sponge *Hemimycale arabica* afforded two new *N*-alkylated hydantoin derivatives, hemimycalins A and B (**2** and **3**), together the previously reported (*Z*)-5-(4-hydroxybenzylidene)-imidazolidine-2,4-dione (**1**). The compounds showed variable antiproliferative and antimicrobial activities.

### 2.2. Structure Elucidation of Compound **1**–**3**

**Figure 1:**
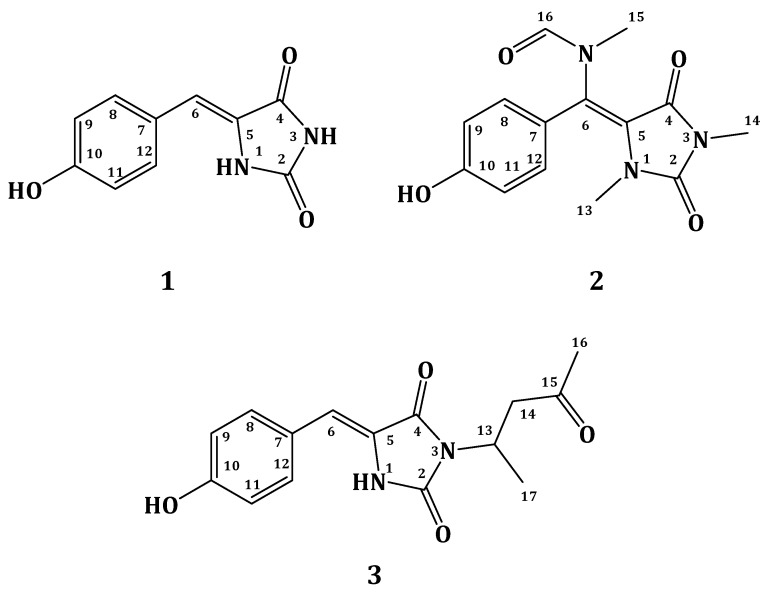
Structures of compounds **1**–**3**.

Compound **1** (Figure 1) was purified as yellow amorphous solid. Its molecular formula C_10_H_8_N_2_O_3_ was deduced from the positive HRESIMS (high-resolution electrospray ionisation mass spectrometry) pseudomolecular ion peak at *m*/*z* 227.0435 [M + Na]^+^. The structure of **1** was assigned as (*Z*)-5-(4-hydroxybenzylidene)-imidazolidine-2,4-dione [18,19] based on complete analysis of its NMR data (Table 1) and by comparison to literature data [18,19]. 

**Table 1:** NMR data and HMBC correlations of compound **1** (DMSO-*d*_6_).

Position	δ_C_	δ_H_ (m, *J* in Hz)	HMBC (H→C) ^a^
2	155.7, qC		
4	165.7, qC		H-6
5	125.3, qC		
6	109.2, CH	6.33 (s)	H-8, H-12
7	123.8, qC		H-9, H-11
8	131.2, CH	7.46 (d, 9.0)	H-6
9	115.6, CH	6.76 (d, 9.0)	
10	158.0, qC		H-8, H-9, H-11, H-12
11	115.6, CH	6.76 (d, 9.0)	
12	131.2, CH	7.46 (d, 9.0)	H-6
N*H*, O*H*		10.0-10.5 (br s)	

^a^ HMBC correlations are from proton(s) stated to the indicated carbons.

**Figure 2:**
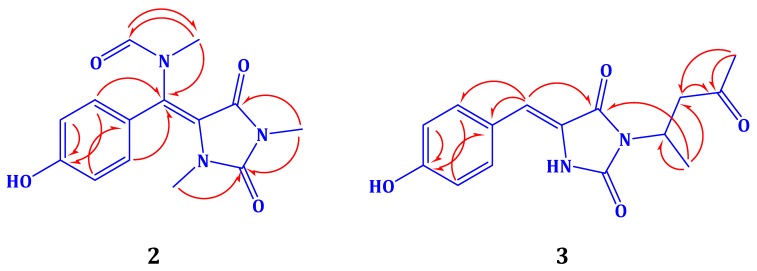
Selected HMBC correlations of compounds **2** and **3**.

Compound **2** (Figure 1) was purified as a yellow amorphous solid with the molecular formula C_14_H_15_N_3_O_4,_ as established from the HRESIMS pseudomolecular ion peak at *m*/*z* 312.0961 [M + Na]^+^, suggesting nine degrees of unsaturation. The ^1^H and ^13^C NMR data of compound **2** (Table 2) showed a similarity to those of **1** (Table 1) with additional signals for two *N*-methyls [20,21,22] and an *N*-methylfromamide moiety [23,24]. The signals at δ_H_/δ_C_ 2.77/31.2, 3.23/29.3, and 2.80/33.5 were assigned as H-13/C-13, H-14/C-14, and H-15/C-15, respectively. The downfield NMR signals at δ_H_/δ_C_ 7.98 (s)/166.1 are characteristic for a formamide methine [23,24]. Furthermore, the ^1^H-^1^H COSY spectrum supported the *p*-substituted phenyl ring. The location of *N*-methyl groups at *N*-1 and *N*-3 was established from HMBC correlations. HMBC cross-peaks (Figure 2) of H-13/C-2, H-14/C-2 (δ_C_ 153.4), and H-14/C-4 (δ_C_ 149.9) supported the location of these *N*-methyl groups. Furthermore, the location of the *N*-methylformamide moiety at C-6 was secured from HMBC correlations of H-15/C-6 (δ_C_ 126.0) H-15/C-5 (δ_C_ 93.8), H-15/C-16 (δ_C_ 166.1), and H-16/C-15 (δ_C_ 33.5) (Figure 1 and Figure 2). Finally, the chemical shift values of the *para*-substituted benzene ring were in good agreement with those of **1** (Table 1). From the above discussion, compound **2** was assigned as *N*-((*E*)-(4-hydroxyphenyl) (1,3-dimethyl-2,5-dioxoimidazolidin-4-ylidene)methyl)-*N*-methylformamide) Figure 1 and Figure 2). To the best of our knowledge, compound **2** is reported here for the first time from a natural source and is considered as a new compound. The generic name hemimycalin A was given to **2**.

**Table 2:** NMR data and HMBC correlations of compound **2** (DMSO-*d*_6_).

Position	δ_C_	δ_H_ (m, *J* in Hz)	HMBC (H→C) ^a^
2	153.4, qC		H_3_-13, H_3_-14
4	149.9, qC		H_3_-14
5	93.8, qC		H_3_-13
6	126.0, qC		H-8, H-12, H_3_-15
7	124.6, qC		H-9, H-11
8	131.1, CH	7.44 (d, 8.4)	
9	115.6, CH	6.75 (d, 8.4)	
10	159.4, qC		H-8, H-9, H-11, H-12
11	115.6, CH	6.75 (d, 8.4)	
12	131.1, CH	7.44 (d, 8.4)	
13	31.2, CH_3_	2.77 (s)	
14	29.3, CH_3_	3.23 (s)	
15	33.5, CH_3_	2.80 (s)	H-16
16	166.1, CH	7.89 (s)	H_3_-15
O*H*		10.88 (br s)	

^a^ HMBC correlations are from proton(s) stated to the indicated carbons.

Compound **3** (Figure 1) showed the molecular formula C_15_H_16_N_2_O_4,_ as established from the pseudomolecular ion peak at *m*/*z* 311.1009 for [M + Na]^+^, requiring nine degrees of unsaturation. The NMR spectra of **3** (Table 3) showed a close similarity to those of **1** (Table 1). Additional signals for the 4-aminopentan-2-one moiety were observed. This was evident for the ^1^H-^1^H COSY non-interrupted spin-coupling system from H_3_-17 (δ_H_ 1.36, *J* = 7.2 Hz) to H-13 (δ_H_ 4.64, m) which further couples with H_2_-14 (δ_H_ 3.35 and 3.00) (Table 3). A three-proton singlet at δ_H_ 2.12 (H_3_-16) together with the ^13^C NMR signal at δ_C_ 208.6 (qC, C-15) are characteristic for a terminal methyl ketone moiety. The HMBC correlations (Figure 2) of H_3_-16/C-15, H_2_-14/C-15 (δ_C_ 208.6, qC), H_2_-14/C-13, H_3_-17/C-13 (δ_C_ 44.4, CH), and H_3_-17/C-14 (δ_C_ 47.0, CH_2_) secured the assignment of this alkyl side chain. The attachment of this alkyl moiety to *N*-3 was secured from the HMBC correlation from H-13 to C-4, completing the assignment of compound **3** (Figure 1 and Figure 2) as (*Z*)-5-(4-hydroxybenzylidene)-3-(4-oxopentan-2-yl) imidazolidine-2,4-dione. To the best of our knowledge, compound **3** is reported here for the first time from a natural source and is considered as a new compound. The generic name hemimycalin B was given to **3**.

**Table 3:** NMR data and HMBC correlations of compound **3** (DMSO-*d*_6_).

Position	δ_C_	δ_H_ (m, *J* in Hz)	HMBC ^a^
2	156.6, qC		H_3_-13
4	166.2, qC		H-6, H-13
5	125.7, qC		H-6
6	112.9, CH	6.52 (s)	H-12
7	125.0, qC		H-6, H-9, H-11
8	132.2, CH	7.33 (d, 8.4)	H-6
9	117.0, CH	6.83 (d, 8.4)	
10	159.9, qC		H-8, H-9, H-11, H-12
11	117.0, CH	6.83 (d, 8.4)	
12	132.2, CH	7.33 (d, 8.4)	
13	44.4, CH	4.64 (m)	H_3_-17
14	47.0, CH_2_	3.35 ^b^ 3.00 (dd, 18.0, 6.0)	H_3_-16, H_3_-17
15	208.6, qC		H_2_-14, H_3_-16
16	29.9, CH_3_	2.12 (s)	H_3_-15
17	18.7, CH_3_	1.36 (d, 7.2)	
N*H*		10.47 (br hump)	

^a^ HMBC correlations are from proton(s) stated to the indicated carbons; ^b^ Overlapped with the H_2_O signal of the solvent.

### 2.3. Biological Activities of the Isolated Compounds

Compounds **1**–**3** were evaluated for their antiproliferative activity against the human cervical carcinoma HeLa cell line as well as for their antimicrobial activity against *E. coli*, *S. aureus*, and *C. albicans*. In the antiproliferative activity evaluation of the HeLa cells (Table 4), compound **1** showed moderate antiproliferative activity against HeLa cells with IC_50_ values of 28.3 μg/mL. The other compounds were weakly active with IC_50_ > 50 μg/mL. In the antimicrobial screen, compounds **1**–**3** displayed good activity against *E. coli* with inhibition zones of 18, 10, and 20 mm, respectively, while these compounds showed inhibition zones of 22, 14, and 20 mm against *C. albicans*. In addition, the compounds were inactive against *S. aureus* (Table 4).

**Table 4:** Antiproliferative and antimicrobial activities of compounds **1**–**3**.

Compound	Antiproliferation Activity (IC_50_, μg/mL)	Antimicrobial Activity Inhibition Zone (mm) at 100 μg/disc		
	HeLa Cell	*S. aureus*	*E. coli*	*C. albicans*
Compound **1**	28.3	NI	18	22
Compound **2**	>50	NI	10	14
Compound **3**	>50	NI	20	20
Paclitaxel ^a^	0.0014	-	-	-
Ciprofloxacin ^b^	-	22	30	-
Ketoconazole ^c^	-	-	-	30

^a^ positive cytotoxic control; ^b^ positive antibacterial control (5 μg/disc); ^c^ positive antifungal control (50 μg/disc); NI = No inhibition.

## 3. Experimental Section

### 3.1. General Experimental Procedures 

IR spectra were measured on a Shimadzu Infrared-400 spectrophotometer (Shimadzu, Kyoto, Japan). Positive mode HRESIMS data were obtained on a Finnigan MAT-312 spectrometer (ThermoFinnigan GmbH, Tokyo, Japan). NMR spectra were obtained in DMSO-*d*_6_ on Bruker Avance 600 spectrometer at 600 MHz for ^1^H NMR and 150 MHz for ^13^C NMR. NMR chemical shifts are expressed in parts per million (ppm) referenced to residual DMSO-*d*_6_ solvent signals (δ_H_ 2.49 for ^1^H and δ_C_ 39.54 for ^13^C). For column chromatography, silica gel (70–230 mesh, Merck, Darmstadt, Germany) was used. Pre-coated SiO_2_ 60 F_254_ plates (Merck, Darmstadt, Germany) were used for TLC. HPLC purification was performed using Prominence Shimadzu HPLC System (Shimadzu Corporation, Tokyo, Japan). 

### 3.2. Biological Materials

**Figure 3:**
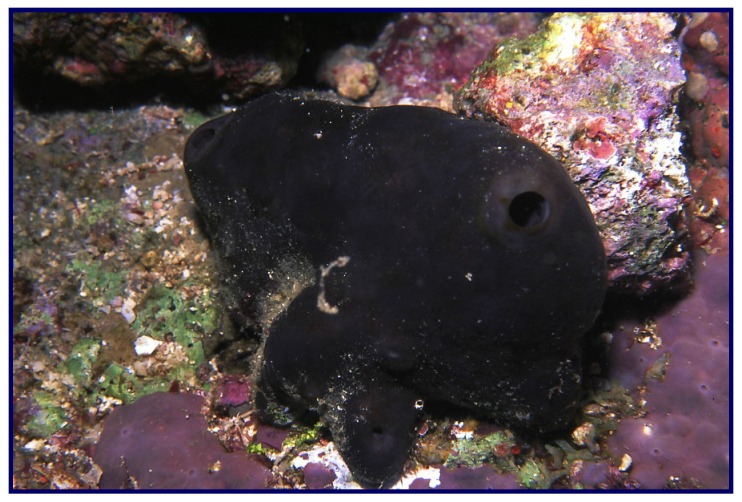
Underwater photograph of the Red Sea sponge *Hemimycale arabica*.

The sponge was collected by hand off the Saudi Red Sea coast of Jazan (Ghurab, north side) (N 17°06′38.0″ E 42°04′01.9″) at depths ranging from 10 to 17 m in May 2013. The sponge (Figure 3) forms an encrusting soft mass of about 1–3 cm thick, covering rocks or dead corals. The outer color is deep bluish with a greenish-yellow interior. The sponge is very soft and easy to cut. The blue surface color is caused by a concentrated pigmented surface layer approximately 150 μm in thickness, which fades to a light beige color on preservation. The skeleton is plumose consisting of parallel loose bundles of thin spicules running from the substratum upwards through the sponge and fanning out at the surface. In between there are many loose spicules. Bundles have a diameter of 30–50 μm and contain 12–20 spicules in cross-section. Siliceous spicules are straight and thin, either strongyles (rounded at both ends) or styles (one end pointed), but otherwise similar in shape and size, ranging from 210–250 × 2–4 μm. These characters conform to the description of the type specimen of *Hemimycale arabica*, with which the present specimen has been compared. A voucher specimen is kept in the collections of the Naturalis Biodiversity Center at Leiden, The Netherlands, registration number ZMA Por. 16634. Another voucher specimen was deposited in the Red Sea Invertebrates Collection of the Department of Natural Products, Faculty of Pharmacy at King Abdulaziz University, under the code number DY-21. The sponge materials were kept frozen at −20 °C until processed.

### 3.3. Purification of Compounds **1**–**3**

The frozen sponge materials (0.78 kg) were cut into small pieces and were extracted with a mixture of CH_2_Cl_2_/MeOH (1:1). The crude extract was partitioned between in 60% MeOH:H_2_O (500 mL) and CH_2_Cl_2_ (3 × 500 mL). The antimicrobial CH_2_Cl_2_ extract (1.5 g) was subjected to a flash silica gel column eluted with *n*-hexane-CH_2_Cl_2_-MeOH gradients to give 11 fractions. All fractions were subjected to the evaluation of their antimicrobial activities. Based on the antimicrobial results, fraction 9, which was eluted with 20% MeOH in CH_2_Cl_2_, was the most active among the tested fractions. Thus, this fraction (125 mg) was subjected to HPLC purification on a semipreparative HPLC column (RP18, 5 μm, ARII Cosmosil, 250 × 10 mm, Waters ) using 25% acetonitrile in water as an eluting solvent with a flow rate of 2 mL/min and a detection at 220 nm to give compounds **1** (45 mg), **2** (11 mg), and **3** (24 mg).

### 3.4. Spectroscopic Data of Compounds **1**–**3**

*(Z)-5-(4-Hydroxybenzylidene) imidazolidine-2,4-dione* *(1)*. Yellow amorphous solid; IR γ_max_ (film) 3385, 1720, 1646, 1593 cm^−1^; NMR data: see Table 1; HRESIMS *m*/*z* 227.0435 (calcd for C_10_H_8_N_2_O_3_Na [M + Na]^+^, 227.0433).

*Hemimycalin A (2)*. Yellow amorphous solid; IR γ_max_ (film) 3374, 1723, 1645, 1592 cm^−1^; NMR data: see Table 1; HRESIMS *m*/*z* 312.0961 (calcd for C_14_H_15_N_3_O_4_Na [M + Na]^+^, 312.0960).

*Hemimycalin B (3).* Yellow amorphous solid; ${\text{[α]}}_{\text{D}}^{20}$ −12 (*c* 0.1 MeOH); IR γ_max_ (film) 3378, 1724, 1643, 1590 cm^−1^; NMR data: see Table 1; HRESIMS *m*/*z* 311.1009 (calcd for C_15_H_16_N_2_O_4_Na [M + Na]^+^, 311.1008.

### 3.5. Biological Evaluation of Compounds **1**–**3**

#### 3.5.1. Determination of the Antimicrobial Activities Using the Disc Diffusion Assay

The *in vitro* antimicrobial activity was evaluated using the disc diffusion method, as previously described [25]. Varieties of test microorganisms were used, including a Gram-positive bacterium (*Staphylococcus aureus* ATCC 25923), a Gram-negative bacterium (*Escherichia coli* ATCC 25922), and yeast (*Candida albicans* ATCC 14053). The adjusted inoculum of each microorganism, equivalent to a turbidity of 0.5 McFarland standards, was streaked separately using sterile swabs over the surface of Muller-Hinton agar plates. Sterile filter paper discs (6 mm diameter) were impregnated with 100 μg of each compound and applied to the inoculated plates. The plates were incubated at 37 °C for 24 h. Solvent control discs were used to determine any solvent effect. Ciprofloxacin (5 μg/disc) was used as an antibacterial standard, while ketoconazole (50 μg/disc) was used as an antifungal standard. The activity of each compound was determined by measuring the diameter of the inhibition zone in mm. The technique was performed in duplicate, and the mean diameter of each inhibition zone was recorded.

#### 3.5.2. Evaluation of Antiproliferative and Cytotoxic Activities against HeLa Cells

The effects of the compounds **1**–**3** on HeLa cell proliferation and cytotoxicity were evaluated using the sulforhodamine B (SRB) assay [26,27,28]. HeLa cells were grown in Basal Medium Eagle (BME) containing Earle’s salts, 10% FBS, and 50 μg/mL gentamycin sulfate. Cells were plated at a density of 2500 cells per well in a 96-well plate and allowed to adhere and grow for 24 h before compounds were added. The compounds were solubilized in DMSO and added to a final DMSO concentration of 1% in both test wells and vehicle controls. The cells were incubated with compounds or vehicle for an additional 48 h. The IC_50_, the concentrations required to cause a 50% inhibition of cell proliferation, was calculated from the log dose response curves. The values represent the average of 3–4 independent experiments, each conducted in triplicate ± SEM. Cytotoxicity was determined by a cell density lower than that measured at the time of drug addition. Paclitaxel was used as a positive control.

## 4. Conclusions

Investigation of the antimicrobial fraction of the organic extract of the Red Sea sponge *Hemimycale arabica* yielded three hydantoin alkaloids (**1**–**3**), including two new ones, hemimycalins A and B (**2** and **3**). Both **2** and **3** represent the first examples of natural *N*-alkylated hydantoin alkaloids from the sponge. Their structures were determined by extensive one-dimensional (1D) and two-dimensional (2D) NMR (COSY, multiplicity-edited HSQC, and HMBC) studies and high-resolution mass spectral determinations. Compound **1** displayed moderate cytotoxic activity against HeLa cells. In addition, the compounds displayed variable antimicrobial activities against different pathogens. 
